# Robust active binocular vision through intrinsically motivated learning

**DOI:** 10.3389/fnbot.2013.00020

**Published:** 2013-11-07

**Authors:** Luca Lonini, Sébastien Forestier, Céline Teulière, Yu Zhao, Bertram E. Shi, Jochen Triesch

**Affiliations:** ^1^Frankfurt Institute for Advanced Studies, Goethe UniversityFrankfurt am Main, Germany; ^2^École Normale Supérieure Cachan BretagneBruz, France; ^3^Department of Electronic and Computer Engineering, HK University of Science and TechnologyHong Kong, China

**Keywords:** active perception, sparse coding, reinforcement learning, robotics, stereo vision, vergence, robustness

## Abstract

The efficient coding hypothesis posits that sensory systems of animals strive to encode sensory signals efficiently by taking into account the redundancies in them. This principle has been very successful in explaining response properties of visual sensory neurons as adaptations to the statistics of natural images. Recently, we have begun to extend the efficient coding hypothesis to active perception through a form of intrinsically motivated learning: a sensory model learns an efficient code for the sensory signals while a reinforcement learner generates movements of the sense organs to improve the encoding of the signals. To this end, it receives an intrinsically generated reinforcement signal indicating how well the sensory model encodes the data. This approach has been tested in the context of binocular vison, leading to the autonomous development of disparity tuning and vergence control. Here we systematically investigate the robustness of the new approach in the context of a binocular vision system implemented on a robot. Robustness is an important aspect that reflects the ability of the system to deal with unmodeled disturbances or events, such as insults to the system that displace the stereo cameras. To demonstrate the robustness of our method and its ability to self-calibrate, we introduce various perturbations and test if and how the system recovers from them. We find that (1) the system can fully recover from a perturbation that can be compensated through the system's motor degrees of freedom, (2) performance degrades gracefully if the system cannot use its motor degrees of freedom to compensate for the perturbation, and (3) recovery from a perturbation is improved if both the sensory encoding and the behavior policy can adapt to the perturbation. Overall, this work demonstrates that our intrinsically motivated learning approach for efficient coding in active perception gives rise to a self-calibrating perceptual system of high robustness.

## 1. Introduction

A number of studies in the last four decades addressed the question of how sensory neurons encode information and showed that neural systems might employ an efficient code to represent incoming data, i.e., a code that exploits redundant information (Attneave, 1954; Barlow, 1961; Field, 1994). The visual system has been a primary target of these studies, where the main result showed that neurons in primary visual cortex (V1) might encode visual information through a *sparse code*, i.e., a code where, at any given moment, only a few neurons out of the entire population fire. A sparse coding strategy has several benefits (Willshaw et al., 1969; Lennie, 2003), including increased memory, less interference between stored patterns and reduced energy consumption, as compared to a dense code (i.e., where many units are simultaneously active). Importantly, when the sparse coding principle is applied to the encoding of natural images (i.e., scenes from nature), it leads to the emergence of basis functions whose structure resemble that of V1 simple cells' receptive fields (Olshausen et al., 1996). The idea of sparse coding has been confirmed by neurophysiological experiments, showing sparse activation of V1 neurons in primates when probed with image sequences of natural stimuli (Weliky et al., 2003) and has been extended to other sensory domains, including the olfactory and auditory domain (Perez-Orive et al., 2002; Smith and Lewicki, 2006). Most studies treated the problem of efficient coding without considering the effects of behavior. The connection between sensory inputs and behavior, commonly referred to as the *perception-action cycle* is important both to (1) understand the development of sensory representations in neural systems as a function of the task performed (Rothkopf et al., 2009) and to (2) design artificial systems, such as robots that autonomously learn and adapt to a changing environment. Indeed, a big technological challenge for such systems is to learn in an efficient and unsupervised way.

We consider this problem in the context of binocular vision. Binocular disparity, the difference between the image projected on left and right retina, is used by organisms with two frontal eyes as a primary depth cue. In order to focus on a point at a certain depth, the two eyes are required to jointly turn inwards or outwards, such that the same object or world feature appears in the center of both images and disparity is nullified. Such type of eye movement is known as *vergence* and represents a fundamental component of visually-guided behavior.

Many approaches to perform vergence in robotic systems employ computer vision techniques to estimate disparity from stereo-images followed by the use of a feedback controller to move the eyes and nullify disparity. These methods are often dependent on pre-defined system parameters and camera calibration. Some methods have used reinforcement learning to autonomously learn vergence control; however, they all require estimating disparity by the use of a pre-defined set of filters (Piater et al., 1999) or a population of disparity-selective neurons (Franz and Triesch, 2007; Wang and Shi, 2010).

In our previous work (Zhao et al., 2012; Lonini et al., 2013c) we have presented a method that autonomously learns how to verge two cameras on a common world feature based on the efficient coding hypothesis. The model makes use of a form of *intrinsic motivation* to learn efficient sensory representations in the perception-action cycle. A sparse coding model learns to encode sensory information using binocular basis functions at different resolutions, while a reinforcement learner generates the camera movement, according to the output of the sparse coding model. Sensory coding and behavior develop in parallel, by minimizing the same cost function: the error between the original stimulus and its reconstruction by the sparse coding model. The rationale behind the approach is that, the more similar left and right images are, the easier they are to encode. Thus, if the actions taken by the reinforcement learning (RL) agent drive the system to perform correct vergence, the reconstruction error will be minimized. Importantly, the reward to the reinforcement learning agent is generated within the system and does not explicitly specify the goal to be attained.

In this paper we show that the joint learning of the sensory and the control part produces a system that is robust with respect to unmodeled disturbances. This is a critical issue for stereo vision systems: for example an insult to the system might cause a displacement of one camera, which in turn modifies the extrinsic parameters (i.e., the relative offset of the two cameras) of the model of the system. We consider four different types of perturbations that we apply to one camera: blur, roll (in-plane rotation), tilt (vertical misalignment), and pan (horizontal misalignment). We show that the system can still learn vergence despite the perturbations. Moreover, when a perturbation is introduced, adapting the bases of the sparse coding models to the changed input statistics improves the performance, as compared to a case where only the policy of the RL agent is adapted and the bases are tuned to unperturbed images. The results underline the importance of adapting both the sensory encoding and the behavior of the system. The use of an intrinsic reward, coupled to an efficient coding of the sensory inputs, allows the model to continuously learn under a multitude of conditions. This self-calibrating property is highly desirable for robotic systems that have to operate in changing environments.

We use the head of the humanoid robot iCub as a test platform. The iCub robot stereo head represents a convenient platform to study active perception, because it replicates the main degrees of freedom of the human head and eyes. We train the model using the iCub simulator and use it to quantitatively assess the performance of the system. We then show that the model also works well on the real robot. The paper is organized as follows: in section 2 we describe the model architecture, the perturbations used and the experimental setup. Section 3 contains the results of the robustness analysis and section 4 discusses the results.

## 2. Materials and methods

In this section, we first provide an overview of the architecture of the vergence control system; then we describe the set of distortions applied to the stereo images, which are used to assess the robustness of the method. Finally we describe the iCub robotic platform and the simulator used to run the experiments.

### 2.1. Model architecture

**Figure 1:**
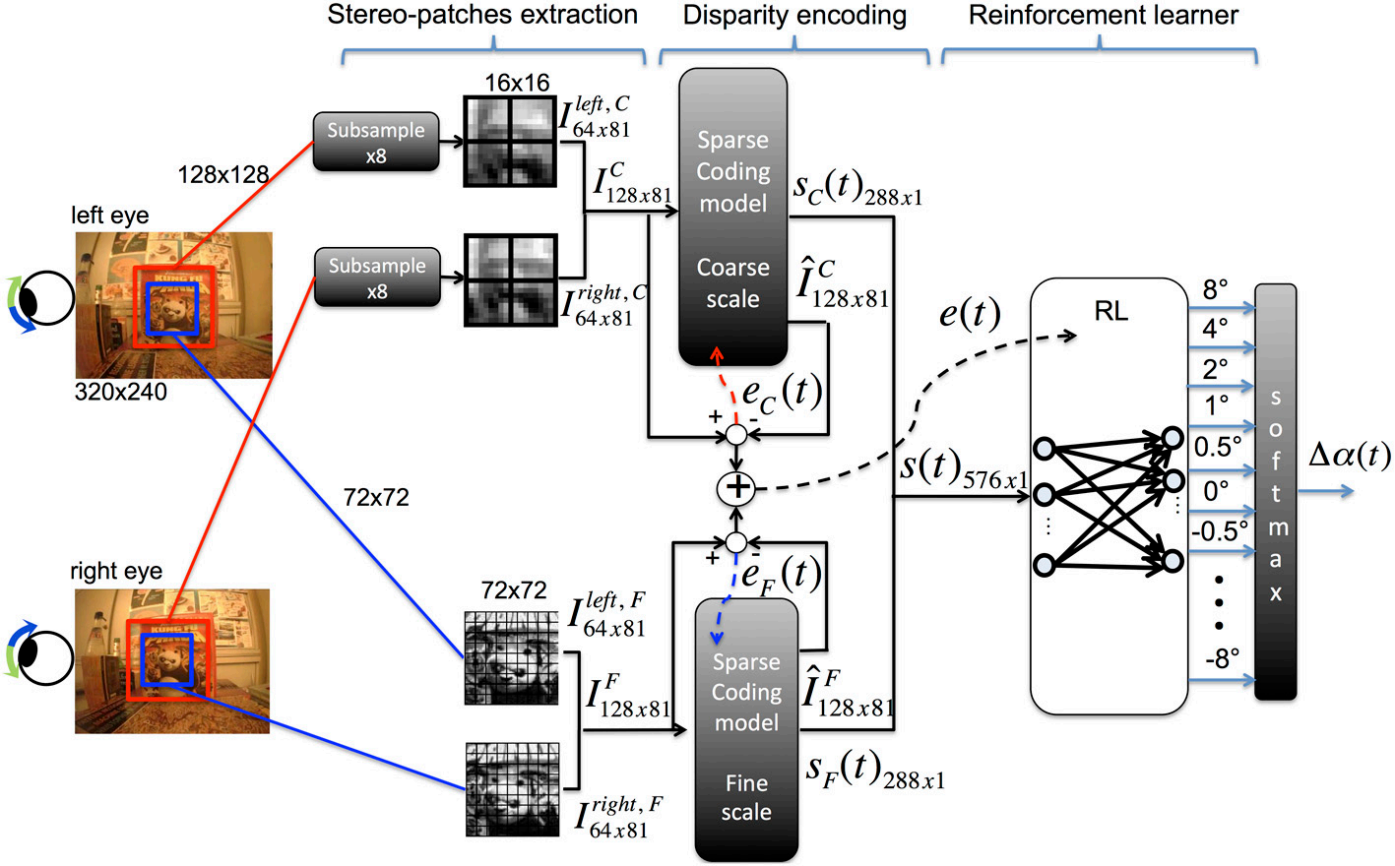
**Architecture of the model for vergence control [adapted from Lonini et al. (2013c)]**.

The vergence control model consists of three main stages (see Figure 1):

- Pre-processing: stereo patches are extracted from the input binocular images and normalized.
- Sensory encoding: two sparse coding models are used to encode the input images at different resolutions.
- Motor control: a reinforcement learning agent generates vergence commands to move the cameras of the robot according to the output of the sparse coding models.

A detailed description of our model architecture has been introduced in (Lonini et al., 2013c). We report here the main elements for the sake of completeness.

#### 2.1.1. Pre-processing

Stereo images are acquired from the cameras of the iCub robot (320 × 240 pixels) and converted to gray-scale. The fixation point is defined to be at the center of each input image. A 128 × 128 pixel image is cut from the center of left and right images (Figure 1, red windows); the image is subsampled to 16 × 16 pixels using a Gaussian pyramid and patches of size 8 × 8 pixels are extracted; this set of patches (receptive fields) corresponds to patches of size 64 × 64 in the original image. The subsampling operation is performed to reduce the computational burden required to train the sparse coding model as well as to learn basis functions at a coarse resolution. To learn basis functions at a fine resolution, patches of size 8 × 8 pixels are extracted from 72 × 72 pixel foveal windows (Figure 1, blue windows), without performing any subsampling. From each foveal window, we extract a total of 81 patches of size 8 × 8 pixels, where patches at the coarse scale are shifted horizontally and/or vertically by multiples of 1 pixel. This ensures that the same number of patches is extracted at each scale. For each scale, each left (right) patch is transformed into a column vector *x^L^_k_* (*x^R^_k_*) and preprocessed to have zero mean and unit norm. Corresponding left and right patches are vertically concatenated to form a stereo-patch *x_k_*, where the first 64 components of *x_k_* correspond to the left patch and the last 64 correspond to the right patch. The subscript *k* indexes the patch within an image.

#### 2.1.2. Sparse coding model

The input to a sparse coding model is a matrix of 81 patches within an input stereo image at a given scale (i.e., coarse or fine). A stereo patch is approximated through the sparse coding model by a linear combination of binocular (stereo) basis functions ϕ. Formally this approximation is expressed by:

(1) $$\left[\begin{array}{c} {\hat{x}}_{k}^{L}\\ {\hat{x}}_{k}^{R} \end{array}\right]=\sum_{i\;=\;1}^{B}a_{i}^{\left(k\right)}\left[\begin{array}{c} \phi_{i}^{L}\\ \phi_{i}^{R} \end{array}\right],$$

where *B* = 288 is the total number of basis functions available in the dictionary of each sparse coding model. In order to ensure sparseness of the representation we allow only 10 coefficients *a_i_* to be non-zero. The sparse coding model is trained to represent the original image as accurately as possible given this sparseness constraint. The total squared reconstruction error over all the stereo-patches, normalized by the energy in the original image measures the loss of information due to the encoding. This is defined by:

(2) $$e=\frac{\sum_{k=1}^{P}\|x_{k}-{\hat{x}}_{k}{\|}^{2}}{\sum_{k=1}^{P}\|x_{k}{\|}^{2}},$$

where *P* is the total number of patches within an image.

Learning occurs online through a two-step procedure: for each patch, a set of coefficients *a_i_* and basis functions ϕ are selected from the basis dictionary using matching pursuit (Mallat and Zhang, 1993), a greedy algorithm that finds a set of bases to represent the input patch. Then, the chosen bases are adapted through gradient descent on the reconstruction error function (Olshausen et al., 1996). Given a foveal window *I*^*j*^(*t*) at time *t* and scale *j* (i.e., coarse or fine), we compute the *B*-dimensional feature vector, *s*_*j*_(*t*), by averaging the squared weighting coefficients over the *P* patches taken from the window:

(3) $$s_{j}(t)=\left[\begin{array}{c} \frac{1}{P}\sum_{k=1}^{P}{\left(a_{1}^{\left(k\right)}\left(t\right)\right)}^{2}\\ \vdots \\ \frac{1}{P}\sum_{k=1}^{P}{\left(a_{B}^{\left(k\right)}\left(t\right)\right)}^{2} \end{array}\right],$$

where *a^(k)^_i_* denotes the coefficient^1^ of basis *i* for patch *k*.

In biological terms, each entry of the state vector models the pooled responses of binocular simple cells (coefficients *a*^(*k*)^_*i*_ for a given *i*) over different locations of the visual field (different patches *k*). The receptive field of a binocular simple cell is represented here by a basis function ϕ_*i*_, which is sensitive to a specific orientation, spatial frequency and disparity. The result of this pooling roughly corresponds to the operation performed by complex cells, which receive inputs from many simple cells at different locations and tuned to the same disparity.

#### 2.1.3. Reinforcement learning

The reinforcement learning agent receives as input the combined feature vector *s*(*t*) from each scale and maps it to a vergence change Δα(*t*). The reward for the agent is the negative sum of the reconstruction errors of the two sparse coding models. The goal of the RL agent is to select actions to maximize the discounted cumulative future reward *R*(*t*)

(4) $$R(t)=\sum_{k=0}^{\infty }-\gamma^{-k}\text{​}\left[e_{C}(t+k)+e_{F}(t+k)\right],$$

where *e*_*C*_ and *e_F_* are the reconstruction errors (2) for the coarse and fine scale sparse coding models, respectively^2^.

The RL architecture we use is the natural actor-critic algorithm as described in Bhatnagar et al. (2009), with an additional regularization factor to keep the weights of the policy bounded. Two linear neural networks (NN) are used to implement the actor (policy) and the critic (value function). The critic network receives as an input the state *s*(*t*) and produces as output the value *V*(*t*) of the current state

(5) $$V(t)=v^{T}(t)s(t),$$

where *v*(*t*) are the weights of the network at time *t* and the superscript ^*T*^ denotes the transpose operator. The policy network maps states to actions and its output layer contains as many neurons as possible actions that the agent can generate. Each action is a relative change Δα(*t*) in the current vergence angle α(*t*). We chose a set *A* of 11 actions, uniformly spaced on a logarithmic scale as *A* = {−8°, −4°, −2°, −1°, −0.5°, 0°, 0.5°, 1°, …, 8°} to allow coarse and fine movements.

The activation *z_a_* of each output neuron at time *t* is computed as

(6) $$z_{a}(t)=\theta_{a}^{T}(t)s(t),$$

where θ_*a*_(*t*) is the vector of weights from the state *s* to action *a* at time *t*.

The probability of choosing action *a* is computed according to a softmax operation on the activation of the output neurons that is:

(7) $$\pi_{a}(s(t))=\frac{\text{exp​}\left(\beta z_{a}(t)\right)}{\sum_{j=1}^{11}\text{exp}\text{​}\left(\beta z_{j}(t)\right)},$$

where β is the inverse of the temperature parameter which controls the amount of exploration vs. exploitation. During training this parameter is set to 1.

### 2.2. Image perturbations

We consider four types of perturbations to assess the robustness as well as the adaptation properties of the model. These perturbations simulate either an unmodeled disturbance or the consequence of an event which causes a change in the extrinsic camera parameters (e.g., a collision). The perturbations are simulated by applying the following transformations to one of the cameras of the robot (we chose the right one):

- Blur: the original image is blurred by applying a rotationally symmetric Gaussian lowpass filter. Three different levels of blur are chosen, corresponding to the following three different combinations of the standard deviation σ and kernel size *S* of the filter reported in Table 1.
- Rotations: We add a constant roll (5°, 15° or 25°), tilt (2°, 6° or 16°) or pan (2° or 4°) angle to the right camera. The roll simulates an in-plane rotation of the camera; the tilt and pan mainly produce, respectively, a vertical and horizontal offset of the right image with respect to the left image. In biological terms, the pan and tilt rotations have a loose analogy with the clinical condition named strabismus, where the gaze direction of one eye is constantly deviated with respect to that of the other eye. In a robotic system this perturbation might occur as a result of an insult to the system.

**Table 1:** **Parameters of the different blur levels**.

**σ** [px]	4	16	32
**S** [px]	8 × 8	32 × 32	64 × 64

**Figure 2:**
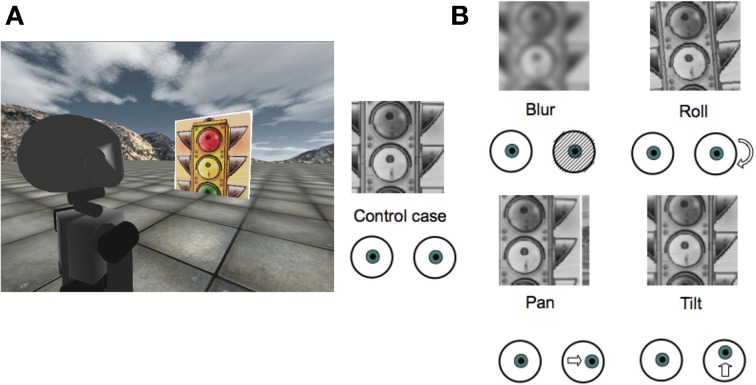
**(A)** A screenshot from the iCub simulator showing the experimental setup; **(B)** Types of perturbations applied to right image from the robot camera (Blur of σ = 4 px; Roll of 5°; Pan of 4°; Tilt of 2°).

The effect of each perturbation is shown in Figure 2B. Details on how to simulate those rotations from the original images are provided in the Appendix. Importantly, since the RL agent can only change the vergence angle, tilt and roll perturbations can not be fully compensated. In contrast, the effect of a pan perturbation can be fully compensated by the model through the controlled degree of freedom. We assess how the model deals with each condition.

### 2.3. Experimental setup

The iCub robot is an open source humanoid robotic platform. The head of the robot (Beira et al., 2006) has a total of six degrees of freedom: three in the neck (pan, tilt, roll) and three in the eyes (independent pan for left and right eye, common tilt). In our setup, we keep the neck of the robot fixed and control anti-symmetrically the pan of the two eyes such as to only modify the vergence angle. In order to accurately quantify the performance of our method, we train the model using the iCub simulator, which provides a controlled environment for extensive testing.

The stereo images acquired from the cameras of the simulated robot have a resolution of 320 × 240 pixels. The focal length is equivalent to 257 pixels which yields a horizontal field of view of ~64°. Thus, a patch at the coarse and fine scale subtends a visual angle of, respectively, 14.2 and 1.8°.

We use a flat square object of side 1 m fronto-parallel to the robot at a varying distance ranging from 0.5 to 2 m (Figure 2A). During training the object distance is varied uniformly within that range every 10 iterations. This range of distances corresponds to vergence angles varying from 8 to 2°. We constrain the maximum vergence angle to be 20°. Similarly, the texture applied on the object is also changed by randomly drawing it from a set of 24 different images. Changing the texture provides the sparse coding model with sufficient statistics about the environmental stimuli to allow a diverse set of basis functions to develop. Training is performed online, where the sparse coding model as well as the RL are both updated at each iteration of the algorithm.

## 3. Results

We first compare how performance changes when a distortion is present, with respect to the control model (i.e., a model trained without any distortion). Each model is trained for 100,000 iterations and performance is measured by the absolute mean vergence error (AME) during training. Since the largest action that the model can take in one step corresponds to a change of 8° in vergence, more than one step may be required to reach the target vergence value. For example, if the current vergence is 20° and the target vergence is 1°, the minimum number of steps required to reach the target vergence is 4 (one possible sequence of actions is −8°, −8°, −2°, −1°). In order to prevent a bias in the estimation of the performance, we only consider the error in the iteration preceding the stimulus change (i.e., the 9th iteration after presentation of a new stimulus). If the new stimulus is introduced at time *t*, the AME is

(8) $$\text{AME}(t)=\frac{1}{N}\sum_{k=-N/2+1}^{N/2}\left|\alpha \text{​}\left(t+9+10k\right)-\alpha^{*}\text{​}\left(t+9+10k\right)\text{​}\right|\;\;\;\;$$

where α^*^ is the target vergence angle for the stimulus and *N* is the size of the averaging window. In our experiments we use a value of *N* = 500 iterations. Since the averaging window is centered on the data point, to compute the AME when there are no previous or subsequent data points available (i.e., *t* < *N*/2 and *t* > *T* − *N*/2, with *T* being the total number of training iterations) we replicate the data point^3^.

**Figure 3:**
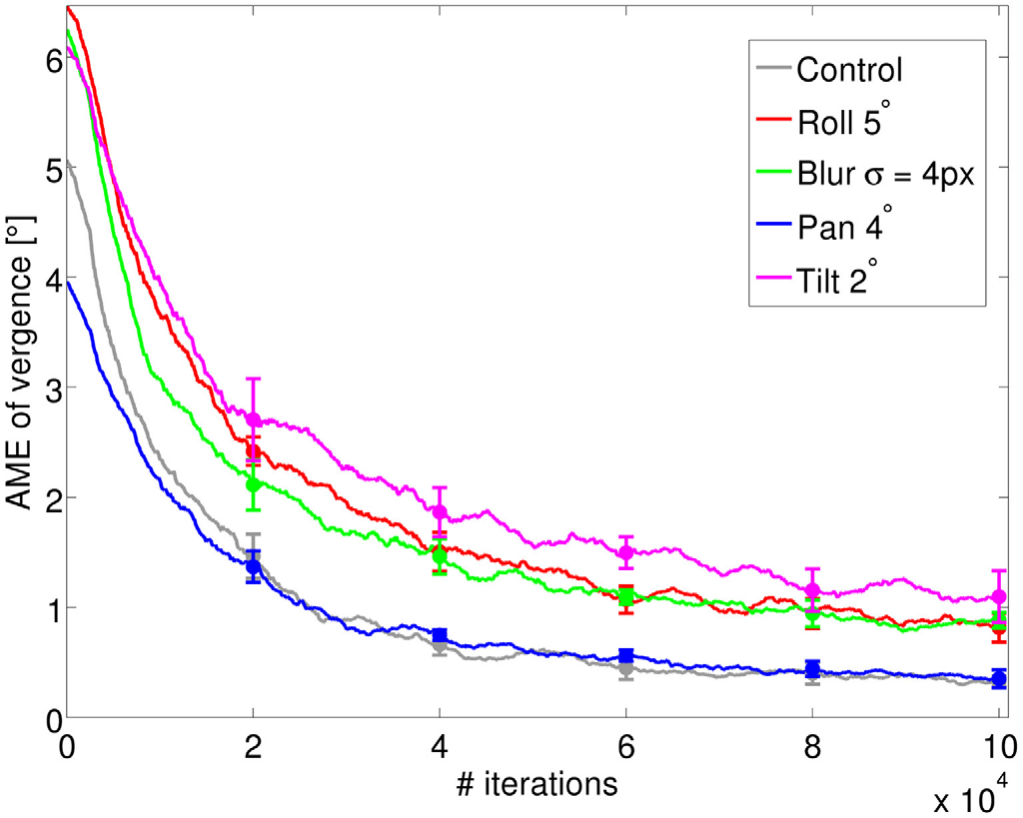
**The AME of vergence during training for the four types of perturbations introduced (blur, roll, pan, and tilt), averaged over 5 simulations**. The control represents the case where no perturbation is applied (gray line). The AME for a random policy is ~7.5° (not shown). Error bars represent one standard deviation.

Figure 3 shows the AME during training for four different perturbations, averaged over five different simulation runs. The level of the perturbation that we use corresponds to the images of Figure 2. As we can see from the decrease of the vergence error, the model can learn to verge under all types of perturbations considered. As a comparison, a random policy for selecting actions would lead to a vergence error of 7.5°. The performance of the system and its final accuracy depend both on the type of perturbation and, as we will show below, on its level. The AME for the control settles at ~0.2° at the end of training. For the pan rotation (horizontal misalignment) the model displays a similar performance. This is because the system can still find a position of zero disparity and maximum redundancy by acting on the vergence angle. The vergence position in this case will correspond to the fixation on a point that is horizontally shifted by 2° with respect to the fixation point of the control case. This vergence position can be reached without any change in the system since our RL agent outputs relative vergence angles. For the other three perturbations the final accuracy is lower compared to the control case. In the case of blur, this is due to the loss of high frequency information. On the other hand, the tilt and roll rotations induce a change in the redundancy of information between left and right image at the vergence position, which affects the performance. However, the final error is ~1°, which shows good learning of the vergence control.

**Figure 4:**
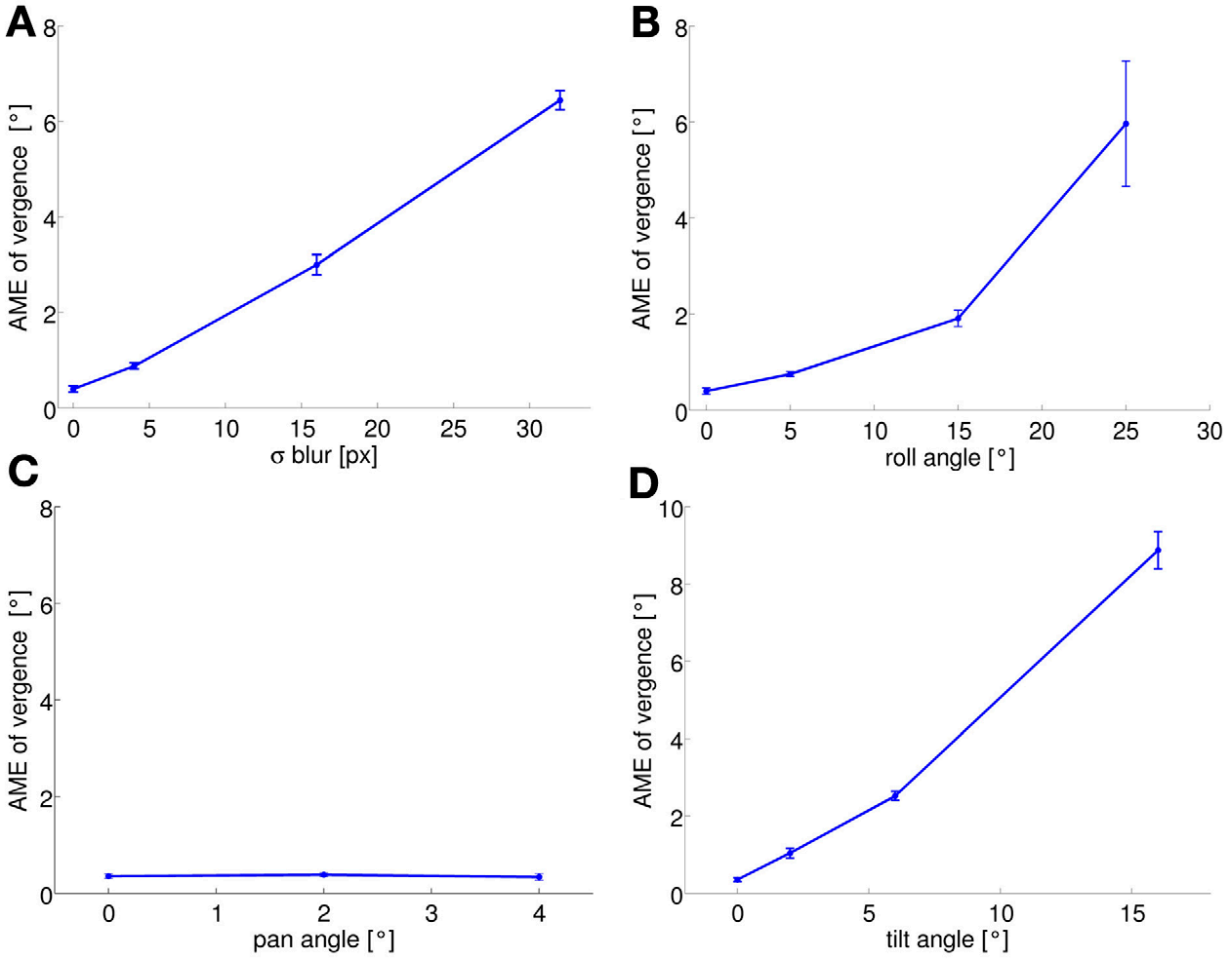
**AME of vergence at the end of training is plotted as a function of the level of each perturbation (average over 5 simulation runs): **(A)** blur; **(B)** roll; **(C)** pan; **(D)** tilt**. AME increases with the level of a perturbation for all types of perturbation, except the pan perturbation (see text). Error bars represent one standard deviation.

As previously mentioned, the performance of the system at the end of learning also depends on the level of the perturbation we introduce. To quantify this performance we run the training phase with different levels of perturbation and observe the final AME of the vergence. Figure 4 shows the results for each type of perturbation, averaged over 5 simulations as before. Again, the AME for a random policy is ~7.5°. As expected, the performances are not affected in the pan rotation case. For the other conditions, the performance degrades as the level of each perturbation increases. In the case of blurred images, the learned policy performs better than a random policy up to values of σ = 16 pixels (AME ~3°). For roll angles up to 15°, the AME is ~2°, indicating that the model can learn vergence, despite the significant rotation between left and right images. For a roll angle of 25° the AME reaches on average 6°. The AME for the tilt perturbation reaches a value of ~2.5° for a tilt angle of 6°, which corresponds to a vertical offset of 18 pixels in the image. When the tilt angle is 16° (vertical offset of 74 pixels), performance degrades drastically and the AME increases to ~8.5°.

In order to assess whether the model can generalize well on new data after training, a test is conducted using a new set of five textures and evaluating the greedy policy. In that case (7) is replaced by

(9) $$\pi_{a}(s(t))=\{\begin{array}{ll} 1 & {\text{if a=argmax}}_{\text{a}}\{z_{a}(t)\}\\ 0 & \text{otherwise} \end{array}\;.$$

**Figure 5:**
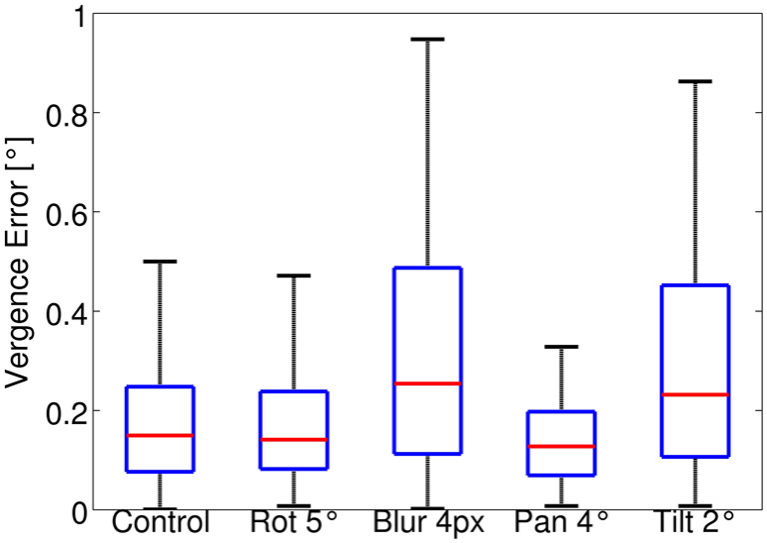
**Box plot of the vergence error in the test session for all types of perturbations considered**. The red line in each box indicates the median; edges of a box are the 25th (*q*_1_) and 75th (*q*_3_) percentiles. The whiskers extend to values up to *q*_3_+1.5(*q*_3_ – *q*_1_). The plotted range extends up to approximately ± 2.7 σ (standard deviation) of the data. Values outside this range are considered outliers and are not plotted.

The stimulus depth is randomly changed between 2 and 0.5 m every 10 iterations and a texture is drawn from the test set every 200 iterations. The test runs for a total of 1000 iterations. The same random sequence is used for all the perturbations. Figure 5 shows a box plot of the vergence error during the test for each case. The median vergence error is used to remove the effect of outliers during the test. For the control case the median of the vergence error is 0.15°. The effect of a roll rotation of 5° is also fully compensated, while the blur (σ = 4 px) and the tilt rotation induce a slightly larger median vergence error, which is ~0.25°. Overall the model performs well in the test sessions for all cases considered. In general, the errors are smaller than that measured at the end of training because the greedy policy is used for testing.

**Figure 6:**
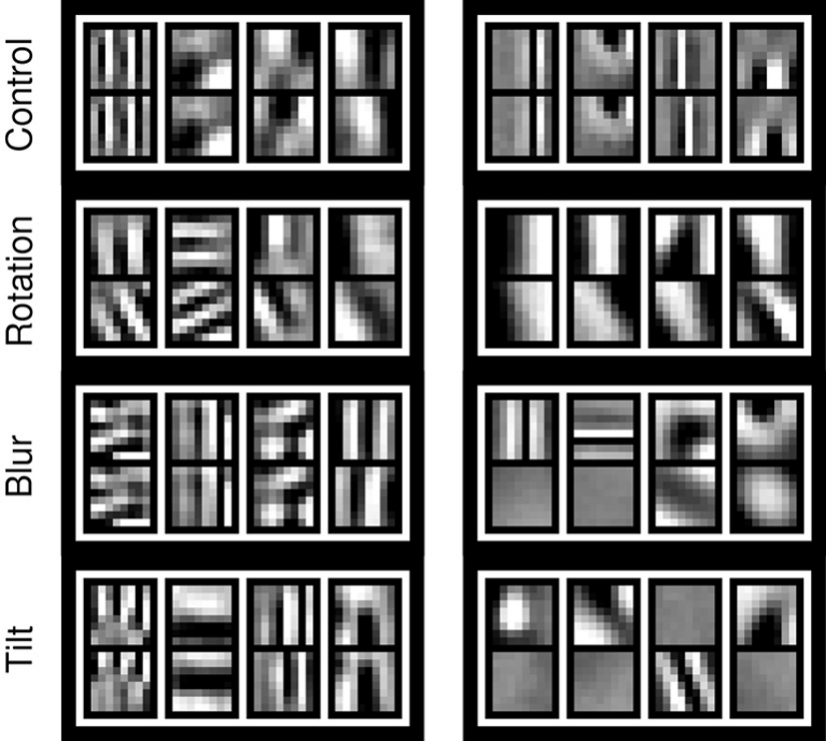
**Example basis functions that emerge at the end of training (100,000 iterations) for coarse (left column) and fine scale (right column)**. Each row corresponds to a different perturbation (Roll of 25°; Blur of σ = 4 px; Tilt of 2°). The pan perturbation case is not considered, as it does not induce any significant change in left and right part of the bases as compared to the control case. See also the video available online for the roll perturbation (Lonini et al., 2013a).

Figure 6 shows example basis functions from the learned dictionaries for each perturbation condition and for each scale. Basis functions are tuned to different orientations and spatial frequencies. Left and right part (vertically concatenated) for bases tuned to zero disparity are identical, while bases tuned to non-zero disparities show a horizontal shift between the left and right part (Figure 6-Control). Each perturbation induces a specific change in the bases that reflects the type of perturbation. The blur condition produces mostly monocular bases at the fine scale, indicated by the fact that the right part is plain. The roll perturbation induces a rotation of the right part with respect to the left, while the tilt rotation produces some bases with a vertical shift between left and right parts, representing vertical disparity.

**Figure 7:**
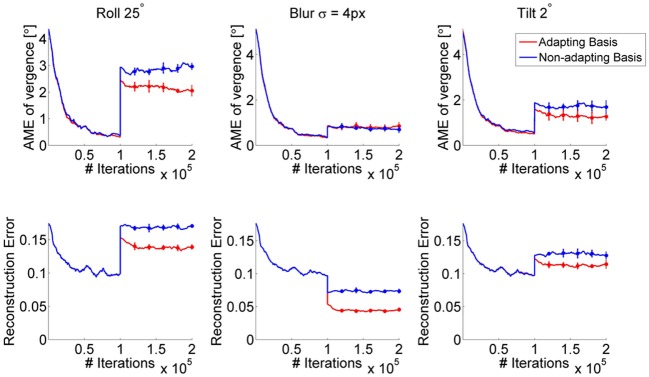
**AME (first row) and reconstruction error of the fine scale sparse coding models (bottom row) when the bases of the sparse coding models are adapted (red) vs. fixed (blue)**. The perturbation occurs after 100,000 iterations. Curves show the average over 5 simulations. Error bars represent one standard deviation.

To assess how adaptation of the bases affects learning of the policy when a perturbation is introduced, we consider the following scenario: we first train a model without any perturbation (control case) for 100,000 iterations. Then, a perturbation is introduced and the model is further trained under either of the following two conditions: first, the bases of the sparse coding models are updated and second, the bases remain fixed as they were before the perturbation. These situations may be roughly analogous to the biological case of an insult to the system occurring either before or after the end of the critical period (Hubel and Wiesel, 1970). In terms of robotics, this could correspond to a perturbation induced by a shock received by the robot, after the system has been trained in an unperturbed scenario. Figure 7 shows the AME during training for three different perturbations (blur, roll and tilt perturbation, first row) as well as the reconstruction error of the sparse coding model for the fine scale (bottom row), under the two conditions. We observe that the AME decreases more for the case where the bases are allowed to change vs. the case when the system uses the same bases learned in the no-perturbation condition (Figure 7, red vs. blue line). Importantly, the policy weights are allowed to change in both cases. Thus, the RL can adapt to the perturbation, even when the same set of basis functions is used. As expected, when the bases are allowed to change, the reconstruction error decreases. This is because the adapted bases can represent the perturbed images better than the original set of bases, trained on unperturbed images; moreover the policy that emerges leads to lower vergence errors, which translates into lower reconstruction errors. Notably, the reconstruction error for the blur case drops in both conditions (adapting and non-adapting bases) because blurring one of the images makes it easier to encode. Also, the AME for the roll perturbation at the onset of the perturbation (~3°) is lower than the AME obtained at the end of training for the same type of perturbation (cfr. Figures 4B, 7). The reason is that the bases trained in absence of the perturbation can still be used to detect disparity, when the perturbation is introduced. A video showing the development of the basis functions, before and after the roll perturbation is introduced, is available online (Lonini et al., 2013a). It can be seen that during exposure to the perturbation, the right part of several basis functions rotates, relatively to the left part.

**Figure 8:**
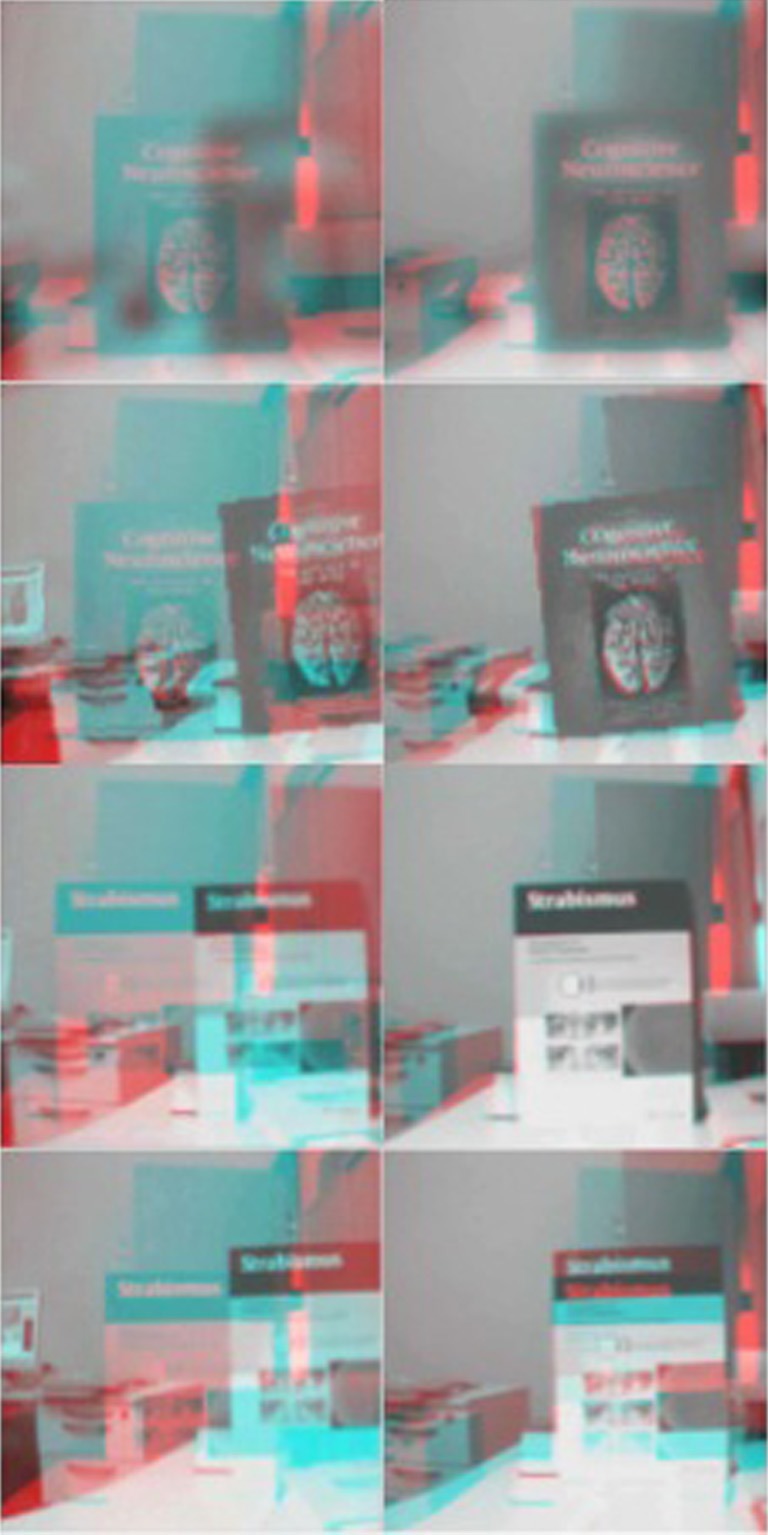
**Test on the real iCub**. Anaglyph of left and right image, before (left) and after (right) vergence is achieved for each perturbation (from top to bottom: Blur of σ = 4 px; Roll of 5°; Pan of 4°; Tilt of 2°). See also online video (Lonini et al., 2013b).

Finally, we test the model trained in the simulator on the real robot to assess the performance when different perturbations are applied. Three sources of uncertainties affect the reliability of the measure of the vergence error on the iCub: 1) the backlash in the DC motors (≤ 1°) that prevents us from accurately measuring the actual vergence angle from the encoder readings; 2) the error in the measure of the distance of the stimulus from the robot; 3) the estimates of the extrinsic camera parameters as well as lens distortions. Figure 8 shows the left and right image anaglyph from the robot cameras before and after vergence is achieved, for all types of perturbations (blur of σ = 4 px; roll of 5°; pan of 4°; tilt of 2°). The model is able to achieve correct vergence under all the perturbations considered. Of notice, the camera parameters of the real iCub differ from that of the simulator. A video of the robot performing the vergence in each condition is available online (Lonini et al., 2013b).

## 4. Discussion

Despite an increasing interest in intrinsic motivations there is still no universally accepted definition. One standpoint is that extrinsic motivations are driven by variables outside of the controller (e.g., battery level, state of the sensors), whereas intrinsic motivations are related to variables within the brain (or controller) of the agent. Thus, intrinsic motivations are driven by epistemic goals, i.e., goals directed to improve the knowledge of the agent, rather than producing a direct change in the world (Baldassarre, 2011). (Zhao et al., 2012) and (Lonini et al., 2013c) have recently proposed a form of intrinsically motivated learning for efficient coding in active perception. They generalize classic notions of efficient coding to movements of the sense organs that facilitate efficient encoding of the sensory data. To this end, a sensory coding model is coupled with a reinforcement learner for controlling the sense organs. The reinforcement learner is rewarded for movements that make the sensory input easier to encode. This approach is closely related to a recent formulation of intrinsic motivations as aiming to maximize compression progress (Schmidhuber, 2009) to create a more compact (and thus interesting) representation of the data. Our system also favors compression progress because achieving a smaller reconstruction error after a vergence command, while using the same amount of neural resources (number of active basis functions), implies that the data are encoded more efficiently. Zhao et al. (2012) and (Lonini et al., 2013c) have shown that in the context of binocular vision, this leads to a fully autonomous learning of disparity representations and accurate vergence control. The system discovers that it is *useful* to properly verge its eyes, because this enables it to encode the sensory data more efficiently.

In this paper we build on this previous work and provide an analysis of the robustness of the approach to various perturbations. We believe that the robustness and self-calibrating properties of a robotic system are a matter of great importance when building autonomous robots capable of adapting to changing environments. We first show that learning occurs under all the perturbations considered and the model performance degrades gracefully with the size of the perturbation. We then compare the condition where the bases (filters) are allowed to adapt when a perturbation is present with the case where they are left unchanged from training on normal images. Adaptation of the bases leads to a more efficient encoding of the input images, which in turns leads the RL to adapt the policy, in a completely unsupervised fashion. Thus, a changed condition in the system, such as a rotation or misalignment of a camera, is automatically handled by our model. A complete compensation of the pan perturbation is obtained as the model controls the vergence angle. Similarly, a full compensation for the tilt and roll perturbation could be achieved if the RL agent was allowed to independently control the tilt and roll angle for each eye.

Previous work addressing the issue of vergence in active stereo vision systems has often relied on computer vision techniques to infer disparity from the stereo pair, and then controlling the stereo cameras through a feedback loop. These methods often require the knowledge of the intrinsic (e.g., focal length and optical centers of the cameras) and the extrinsic (relative position of the two cameras) parameters of the cameras. Examples include cepstral or zero-disparity filters (Olson and Coombs, 1991), correlation-based methods (Capurro et al., 1997) and feature matching (Hansen and Sommer, 1996). Reinforcement learning has been used to learn vergence, by using as reward the disparity estimated through feature matching (Piater et al., 1999) or by a population of disparity-tuned neurons (Franz and Triesch, 2007; Wang and Shi, 2010). The main limitation of these approaches is that the disparity filters are not learned from the data. Importantly, to our knowledge, there is no work that is directly addressing the robustness of a vergence control method to image distortions.

Our model provides a way to autonomously adapt both the sensory representation as well as the control of the behavior by the simultaneous learning of the two systems. The proposed method can be extended to other domains, such as the learning of smooth-pursuit behavior, which is currently under development. Future work should address whether this new framework for efficient coding in active perception can be further extended to other sensory modalities and what insights into the biology of active perception it provides.

### Conflict of interest statement

The authors declare that the research was conducted in the absence of any commercial or financial relationships that could be construed as a potential conflict of interest.

## Footnotes

^1^For the convenience of reading, we drop the index *j* indicating that the coefficients *a_i_* depend on the scale.

^2^Maximizing (4) corresponds to minimizing the total reconstruction error.

^3^For example we assume α(*t*′) = α(0) if *t*′ < 0 .

## Appendix

We describe here how the perturbations are generated. A rotation of a camera induces a projective transformation of the image. To simulate our pan, tilt and roll perturbations we thus compute this transformation, also called *homography*. Formally, the homographic image transformations H, is computed by:

(10) $$H=KRK^{-1},$$

where *K* is a 3 × 3 matrix, containing the camera intrinsic parameters (focal length and image center coordinates) and *R* is a 3 × 3 matrix containing the three angles of rotation of the camera (pan, tilt, in-plane rotation^4^) (Faugeras, 1993). The forms of K and R are the following:

(11) $$K=\left[\begin{array}{ccc} f & 0 & c_{x}\\ 0 & f & c_{y}\\ 0 & 0 & 1 \end{array}\right]\text{​},$$

(12) $$R=\text{ ​}\left[\begin{array}{ccc} c(r_{y})c(r_{z}) & \text{​}-\text{​}c(r_{y})s(r_{z}) & s(r_{y})\\ c(r_{x})s(r_{z})+s(r_{x}) & c(r_{x})c(r_{z})- & -s(r_{x})c(r_{y}))\\ \;\sin(r_{y})c(r_{z}) & \;s(r_{x})s(r_{y})s(r_{z}) & \\ c(r_{x})s(r_{y})c(r_{z})+ & c(r_{x})s(r_{y})s(r_{z})+ & c(r_{x})c(r_{y})\\ \;s(r_{x})\sin(r_{z}) & \;+c(r_{z})s(r_{x}) & \end{array}\right]\text{​},$$

where *c_x_* and *c_y_* are the image center coordinates, *f* is the focal in pixel values and *c*() and *s*() denote, respectively, the cosine and sine operation. *r_x_*, *r_y_*, and *r_z_* indicate the tilt, pan and roll angle, respectively. We thus simulate those rotations by applying the homographic transformation to the acquired images. For each pixel of coordinates [*uv*] of the original image, the corresponding position after the perturbation would be [*u*′ *v*′1]^⊤^ = *H*[*uv*1]^⊤^. Cubic interpolation is used to compute the pixel values at integer pixel coordinates. Remember that the system has no knowledge about the perturbation nor the camera parameters, and the steps described here are only used to simulate a perturbation in one of the cameras. Also, as the result of a rotation is a relative misalignment of the two cameras, it is actually irrelevant whether the rotation is applied to one or both cameras to demonstrate the ability of the system to correct for a perturbation.
